# Comparative analysis of chloroplast genomes of three medicinal *Carpesium* species: Genome structures and phylogenetic relationships

**DOI:** 10.1371/journal.pone.0272563

**Published:** 2022-08-05

**Authors:** Xingyu Shi, Wenfen Xu, Mingxiang Wan, Qingwen Sun, Qiyu Chen, Chao Zhao, Kaifen Sun, Yanxia Shu

**Affiliations:** 1 College of Pharmacy, Guizhou University of Traditional Chinese Medicine, Guiyang, China; 2 First Affiliated Hospital of Guizhou University of Traditional Chinese Medicine, Guiyang, China; Central University of Punjab, INDIA

## Abstract

*Carpesium* (Asteraceae) is a genus that contains many plant species with important medicinal values. However, the lack of chloroplast genome research of this genus has greatly hindered the study of its molecular evolution and phylogenetic relationship. This study used the Illumina sequencing platform to sequence three medicinal plants of the *Carpesium* genus: *Carpesium abrotanoides*, *Carpesium cernuum*, and *Carpesium faberi*, obtaining three complete chloroplast genome sequences after assembly and annotation. It was revealed that the three chloroplast genomes were typical quadripartite structures with lengths of 151,389 bp (*C*. *abrotanoides*), 151,278 bp (*C*. *cernuum*), and 151,250 bp (*C*. *faberi*), respectively. A total of 114 different genes were annotated, including 80 protein-coding genes, 30 tRNA genes, and 4 rRNA genes. Abundant SSR loci were detected in all three chloroplast genomes, with most composed of A/T. The expansion and contraction of the IR region indicate that the boundary regions of IR/SC are relatively conserved for the three species. Using *C*. *abrotanoides* as a reference, most of the non-coding regions of the chloroplast genomes were significantly different among the three species. Five different mutation hot spots (*trnC-GCA-petN*, *psaI*, *petA-psbJ*, *ndhF*, *ycf1*) with high nucleotide variability (Pi) can serve as potential DNA barcodes of *Carpesium* species. Additionally, phylogenetic evolution analysis of the three species suggests that *C*. *cernuum* has a closer genetic relationship to *C*. *faberi* than *C*. *abrotanoides*. Simultaneously, *Carpesium* is a monophyletic group closely related to the genus *Inula*. Complete chloroplast genomes of *Carpesium* species can help study the evolutionary and phylogenetic relationships and are expected to provide genetic marker assistance to identify *Carpesium* species.

## Introduction

The family Asteraceae is the most differentiated dicotyledons with about 1,479 genera and 21,105 species distributed worldwide, except for the Antarctic region [1]. *Carpesium* is a genus of the Asteraceae family with beneficial medicinal value. About 21 species globally are majorly distributed in the Eurasian continent [2]; 17 species and 3 variety species found in China, mainly distributed in the Southwest of China [3]. The genus of the *Carpesium* plants, such as *C*. *abrotanoides*, *C*. *cernuum*, and *C*. *divaricatum* has been widely used as a folk medicine in treating mumps, folliculitis, toothache, colds, and fever [4]. Pharmacological and chemical studies have confirmed that they contain sesquiterpenoids with antibacterial, anti-inflammatory, antimalarial, antitumor, and antioxidant effects [5]. Among these species, *C*. *abrotanoides* is the most widely used longest in history as an herbal medicine. In China, the fruit of *C*. *abrotanoides* is called “He Shi,” possessing antiparasitic properties and eliminating its accumulation [5, 6]. Moreover, its aerial parts are often used to treat bruises and fever [7]. Additionally, the *C*. *cernuum* and *C*. *faberi* are also used as medicinal plants by folks to treat lymph node nuclei, mastitis, fever, sore throat, toothache, blood stranguria, and other diseases [2, 8].

Morphological similarity between plants in *Carpesium* has led to confusion in the base source of folk medicine, affecting the safety and efficacy of medicines obtained from its species to a certain extent. However, current research on this genus is focused on their active chemical components and pharmacological activities. The taxonomic identification of its species is still based on morphological studies, thus leading to inaccurate interspecific identification of the species within the genus. Meanwhile, due to the lack of abundant genetic marker information, research in understanding the phylogenetic position and genetic diversity of the *Carpesium* is still lacking. Therefore, it is necessary to establish more discriminative genetic markers to analyze and discuss the interspecific relationship of this genus and its position in Asteraceae to provide a reliable basis for the genetic identification of medicinal materials.

The chloroplast (cp) is an organelle with a bilayer membrane structure that originates from symbiotic cyanobacteria cells [9, 10] capable of releasing oxygen to convert solar energy into carbohydrates through photosynthesis, aimed at sustaining its life [11]. Cp also plays a vital role in amino acid and lipid synthesis metabolism [12]. In 1986, the whole cp genome of tobacco was sequenced and annotated, and currently, various cp genomes have been reported [13]; therefore, many researchers have been attracted to devote themselves to studying cp genomes of plants. In many angiosperms, the cp genome is usually a quadruplex, consisting of two inverted repeat regions (IRs), a large single-copy region (LSC), and a small single-copy region (SSC), with the IR regions separating the LSC and SSC regions [14]. Generally, cp varies in size from 120 to 180 kb [15], 60 to 130 kb encoding genes mainly involved in photosynthesis and other metabolic processes [10]. Compared to the nuclear genome, the cp genome has a haploid inheritance, conserved structure, smaller genome, and slow mutation rate [16–18], making it an ideal model for molecular identification of species, genetic diversity studies, and revealing phylogenetic relationships [19–21]. Recently, *Artemisia*, *Panax*, *Physalis*, *Paeonia*, *Salvia*, and other genera’s complete cp genome data have been used to identify highly differentiated regions and make phylogenetic inferences, eventually providing a reference for identification and phylogenetic studies of these species [22–26].

Given this, we sequenced and annotated the whole cp genome of *C*. *abrotanoides*, *C*. *cernuum*, and *C*. *faberi* to explore the relationships among the *Carpesium* species. Then, simple sequence repeats (SSRs), interspersed repeated, IR expansion and contraction were investigated, and mutation hot spots were screened. Additionally, a phylogenetic tree was constructed using the whole cp genomes of 38 species of Asteraceae. This study explored the genetic differentiation and structural characteristics of the genus *Carpesium* and its developmental relationships in Asteraceae at the molecular level. It also provides the basis for elucidating the evolutionary process of the genus *Carpesium*, revealing its phylogenetic relationship, and identifying species of the genus.

## Materials and methods

### Collection of plant materials, DNA extraction, and sequencing

The fresh leaves of *C*. *abrotanoides* and *C*. *faberi* were collected from Longli County, Guizhou Province, China, whereas *C*. *cernuum* were collected from Pingba County, Guizhou Province, China. Samples were immediately frozen in liquid nitrogen and stored at −80°C. According to the manufacturer’s instructions, the whole DNA samples were extracted from fresh leaves using an EZNA Plant DNA extraction kit (OMEGA, USA). The quality and quantity of extracted DNA were measured using NanoPhotometer spectrophotometer (IMPLEN, USA) and Qubit 2.0 Fluorometer (Life Technologies, USA), respectively. The genome was sequenced by the Illumina NovaSeq Sequencing System to generate paired-end 2×150 bp reads, and about 7.06 Gb (*C*. *abrotanoides*), 5.48 Gb (*C*. *cernuum*), and 5.63 Gb (*C*. *faberi*) raw data were obtained.

### Cp genome assembly, annotation

Trimmomatic [27] was applied to filter the raw data. Next, NOVOPlasty [28] was adopted to assemble the cp genome, then Gap Close [29] repaired the inner gaps. Finally, the reference genome of *C*. *abrotanoides* was used for correcting the positions and directions of the four cp regions (LSC/IRa/SSC/IRb). The genomes were annotated with manual correction by the CpGAVAS2 [30] and were determined to obtain the complete cp genome sequence. Whole cp genome maps were drawn with the CHLOROPLOT [31]. The annotated genome sequence was submitted to GenBank (with accession numbers: OM302256, OM302257, and OM302258).

### Analysis of codon usage and repeat sequence

MEGA7 [32] was applied to analyze the synonymous codon and relative synonymous codon usage (RSCU) of the three *Carpesium* species. MISA determined the SSR according to Beier et al. (2017) [33] with the following settings: ten repeat units for mononucleotide SSRs, five repeat units for dinucleotide SSRs, four repeat units for trinucleotide SSRs, and three repeat units for tetranucleotide, pentanucleotide, and hexanucleotide repeats. The interspersed repeated analysis was performed using REPuter [34], including the forward repeat (F), reverse repeat (R), complement repeat (C), and palindromic repeat (P), with parameters set at minimal repeat size 30 bp, and 90% sequence identity (hamming distance 3).

### Comparative genome analysis and sequence variation

The boundary information of the four regions (IR, LSC, and SSC areas) of cp genomes was visualized using IRscope according to Amiryousefi et al. (2018) [35]. The three whole cp genomes were compared using the online genome analysis program mVISTA [36], whereas *C*. *abrotanoides* was used as a reference in the Shuffle-LAGAN mode. According to the method of Katoh et al. (2005) [37], MAFFT was used to compare the complete cp genome sequences of 3 species of the genus *Carpesium*, then DNAsp v.6.10 [38] was used for sliding window analysis with a step length of 200 bp and window length of 600 bp.

### Phylogenetic analysis

The three sequenced cp genomes of *Carpesium* and the whole cp genomes of the 35 species (using *Taraxacum mongolicum*, *Taraxacum officinale*, and *Lactuca sativa* as outgroups) were retrieved from the NCBI database for constructing a phylogenetic tree (S1 Table). MAFFT was then applied to align the complete cp genomes of all species with a manual correction [37]. The best nucleotide substitution model was tested with the built-in ModelFinder in IQ-tree. The IQ-tree was then used to construct the maximum likelihood (ML) tree with 1,000 bootstrap replicates [39–41].

## Results

### Characteristics and structure of cp genome

**Fig 1:**
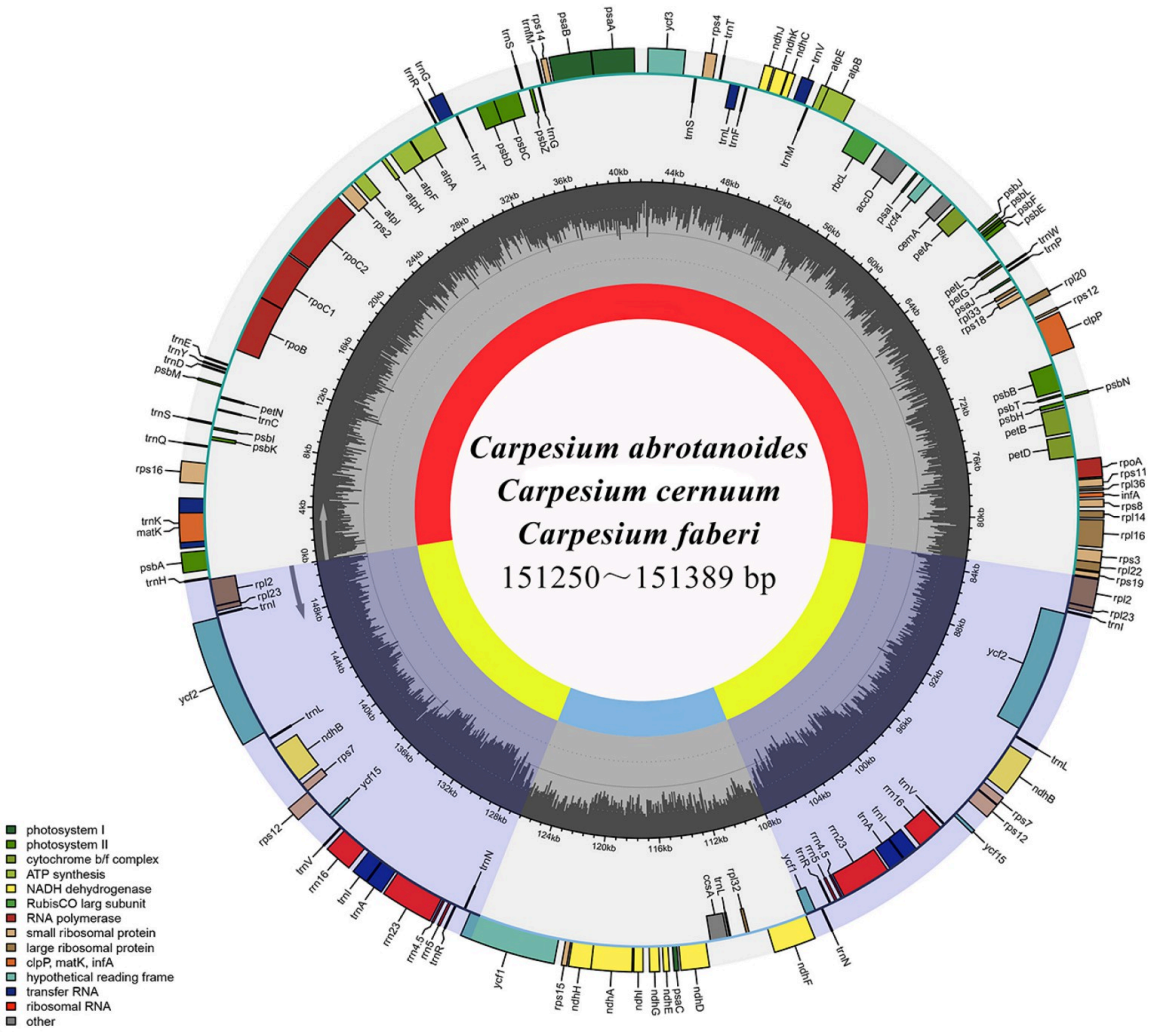
Cp genome mapping of three *Carpesium* species. The inner genes are transcribed clockwise, and the outer genes are transcribed counterclockwise. Different colors indicate genes with different functions. The light black of the inner circle indicates GC content, and dark gray indicates AT content.

The full length cp genomes were 151,389 bp, 151,278 bp, and 151,250 bp for *C*. *abrotanoides*, *C*. *cernuum*, and *C*. *faberi*, respectively. Similar to most angiosperms, the *Carpesium* cp genomes also appeared with a typical quadripartite structure, distributed in one LSC region (82,915 bp–83,059 bp) and one SSC region (18,426 bp–18,447 bp) separated by a pair of inverted repeats (IRa and IRb; 49,004 bp) (Fig 1; Table 1). The overall GC content of the three plants was the closest, ranging from 36.6% to 36.7%. The GC content of the IR regions (43%) was higher than that of the LSC and SSC regions (35.7%–35.9% and 31.2%–31.3%), respectively (Table 1).

**Table 1:** Basic characteristics of the cp genomes of three *Carpesium* species.

		*C*. *abrotanoides*	*C*. *cernuum*	*C*. *faberi*
Genome size (bp)		151,389	151,278	151,250
LSC length (bp)		83,059	82,927	82,915
SSC length (bp)		18,426	18,447	18,431
IR length (bp)		49,904	49,904	49,904
Number of genes (total /unique)		132/114	132/114	132/114
protein-coding genes (CDS) (total/in IR)		87/7	87/7	87/7
rRNA (total/unique)		8/4	8/4	8/4
tRNA (total/unique)		37/30	37/30	37/30
genes duplicated in IR		18	18	18
GC content (%)	Total (%)	37.6	37.7	37.7
	LSC (%)	35.7	35.9	35.9
	SSC (%)	31.2	31.3	31.3
	IR (%)	43	43	43
protein-coding genes (CDS) (% bp)		51.74	51.97	51.97
rRNA genes (% bp)		5.98	5.98	5.98
tRNA genes (% bp)		1.84	1.84	1.84
GenBank accession number		OM302256	OM302256	OM302256

Complete cp genome of *C*. *abrotanoides*, *C*. *cernuum*, and *C*. *faberi* encoded 132 genes. Among them, 114 genes were unique, including 80 protein-coding genes, 30 transfer RNA (tRNA), and 4 rRNA genes. Additionally, one gene (*ycf1*) was annotated as pseudogenes (Table 2). Furthermore, among the 114 genes, 18 genes contained introns (12 protein-coding genes and 6 tRNAs genes), among the 15 genes (*atpF*, *ndhA*, *ndhB*, *petB*, *petD*, *rpl2*, *rpl16*, *rps16*, *rpoC1*, *trnA-UGC*, *trnG-UCC*, *trnI-GAU*, *trnK-UUU*, *trnL-UAA*, and *trnV-UAC*) contained one intron, and the 3 genes (*rps12*, *ycf3*, and *clpP*) contained two introns. Four of these genes (*trnA-UGC*, *trnI-GAU*, *ndhB*, and *rpl2*) appeared in both IR regions, whereas one gene (*ndhA*) was in the SSC region (S2 Table).

**Table 2:** Genetic composition of the cp genomes of three *Carpesium* species.

Category of genes	Group of gene	Gene name	Number
Genes for photosynthesis	Subunits of photosystem I	*psaA*, *psaB*, *psaC*, *psaI*, *psaJ*	5
	Subunits of photosystem II	*psbA*, *psbB*, *psbC*, *psbD*, *psbE*, *psbF*, *psbH*, *psbI*, *psbJ*, *psbK*, *psbL*, *psbM*, *psbN*, *psbT*, *psbZ*	15
	Subunits of ATP synthase	*atpA*, *atpB*, *atpE*, atpF*, *atpH*, *atpI*	6
	Subunits of NADH-dehydrogenase	ndhA*, *ndhB*(×2)*, *ndhC*, *ndhD*, *ndhE*, *ndhF*, *ndhG*, *ndhH*, *ndhI*, *ndhJ*, *ndhK*	12
	Subunits of cytochrome b/f complex	*petA*, petB*, petD*, *petG*, *petL*, *petN*	6
	Subunit of rubisco	*rbcL*	1
Self-repilcation	Large subunit of ribosome	*rpl2*(×2)*, *rpl14*, rpl16*, *rpl20*, *rpl22*, *rpl23*(×2), *rpl32*, *rpl33*, *rpl36*	11
	Small subunit of ribosome	*rps11*, *rps12*(×2), *rps14*, *rps15*, rps16*, *rps18*, *rps19*, *rps2*, *rps3*, *rps4*, *rps7*(×2), *rps8*	14
	DNA-dependent RNA polymerase	*rpoA*, *rpoB*, rpoC1*, *rpoC2*	4
	Ribosomal RNAs	*rrn4*.*5S*(×2), *rrn5S*(×2), *rrn16S*(×2), *rrn23S*(×2)	8
	tRNA genes	*trnA-UGC*(×2)*, *trnC-GCA*, *trnD-GUC*, *trnE-UUC*, *trnF-GAA*, *trnfM-CAU*, *trnG-GCC*, trnG-UCC*, *trnH-GUG*, *trnI-CAU*(×2), *trnI-GAU*(×2)*, trnK-UUU*, *trnL-CAA*(×2), trnL-UAA*, *trnL-UAG*, *trnM-CAU*, *trnN-GUU*(×2), *trnP-UGG*, *trnQ-UUG*, *trnR-ACG*(×2), *trnR-UCU*, *trnS-GCU*, *trnS-GGA*, *trnS-UGA*, *trnT-GGU*, *trnT-UGU*, *trnV-GAC*(×2), trnV-UAC*, *trnW-CCA*, *trnY-GUA*	37
Other genes	Translation initiation factor	*infA*	1
	Subunit of acetyl-CoA-carboxylase	*accD*	1
	c-type cytochrome synthesis gene	*ccsA*	1
	Envelop membrane protein	*cemA*	1
	Protease	*clpP* **	1
	Maturase	*matK*	1
Genes with unknown function	Conserved open reading frames	*ycf1*^a^, *ycf2*(×2), *ycf4*, ycf3**, *ycf15*(×2)	7

(×2): Two gene copies in IRs;

*: gene containing one intron;

**: gene containing two introns;

^a^: pseudogene.

### Codon usage

**Fig 2:**
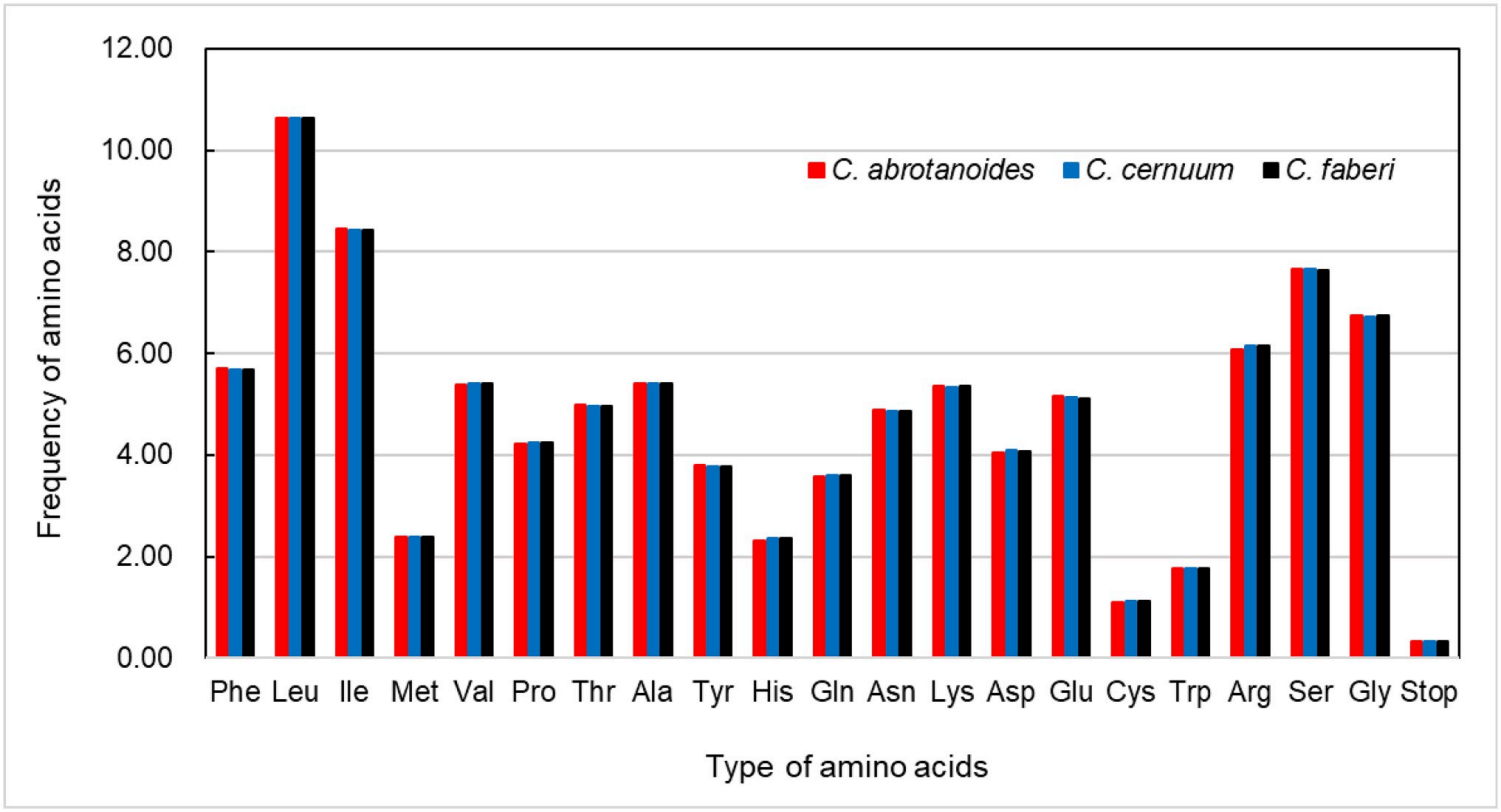
Comparison of amino acid frequencies in the cp genomes of three *Carpesium* species.

Amino acid frequency analysis and RSCU showed high similarities among the species. The protein-coding sequences of the *C*. *abrotanoides*, *C*. *cernuum*, and *C*. *faberi* cp genomes consisted of 26,112, 26,205, and 26,203 codons, respectively (S3 Table). The percentage of the coded amino acids are presented in increasing order as follows; Cysteine (1.11%), Isoleucine (8.42%–8.46%), and Leucine (10.62–10.64%) (Fig 2). Tyagi et al. (2020) [42] reported that the Leucine had the highest, whereas the Cysteines had the lowest abundance of amino acids in other angiosperm cp genomes. In the cp genomes of the three genera, the codon AUG (Methionine) and UGC (Tryptophan) were unbiased with RSCU = 1.00. These two amino acids had no preference because they were encoded using one codon. Additionally, the codons of other amino acids exhibited significant differences. In contrast, the different codons except the UUG containing A or T were the most preferred codon in encoding amino acids with RSCU > 1.

### Identification of SSRs and repeat sequences

**Fig 3:**
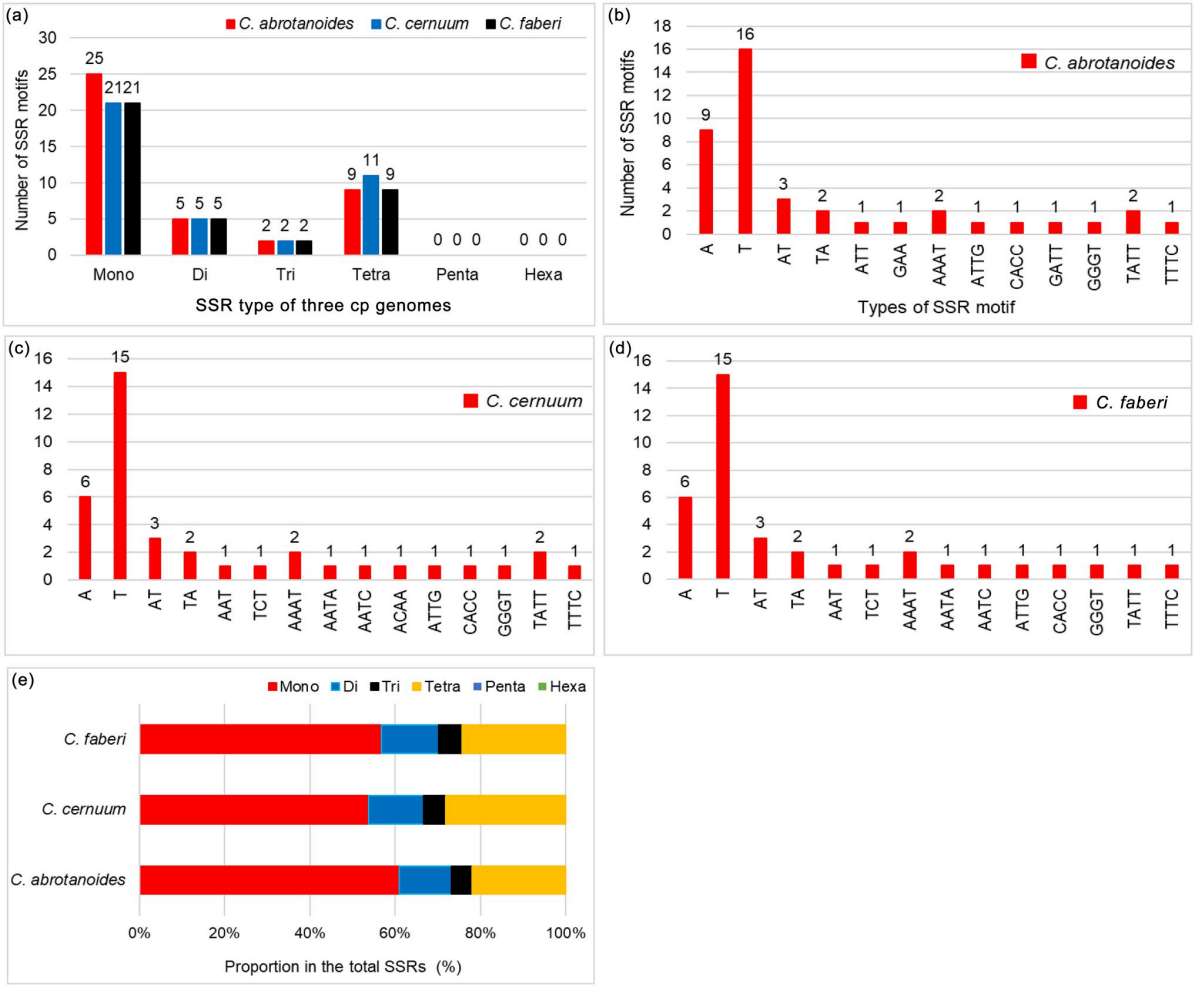
Number and proportion of SSRs in the cp genomes of three *Carpesium* species. (a) The number of different SSR types. (b-d) The frequencies of different SSR types of three *Carpesium* species. (e) The proportion of different SSR types.

MISA detected 41 SSRs in *C*. *abrotanoides*, 39 in *C*. *cernuum*, and 37 in *C*. *faberi* (mononucleotide, dinucleotide, trinucleotide, and tetranucleotide as shown in S4 Table and Fig 3a). Among the three species, we found that the content of mononucleotide A or T homopolymers in the 4 SSR types is the highest, which illustrated that SSRs usually comprises poly-A and poly-T, but rarely tandem guanine (G) and cytosine (C), thereby contributing to the AT abundance in the cp genome (Fig 3b–3d). Furthermore, mononucleotide repeats (53.85%–60.98%) were the most frequent, whereas the trinucleotide repeats (4.88%–5.41%) were the least (Fig 3e), showing that the mononucleotide repeats made more contribution to genetic variations than other SSRs.

**Fig 4:**
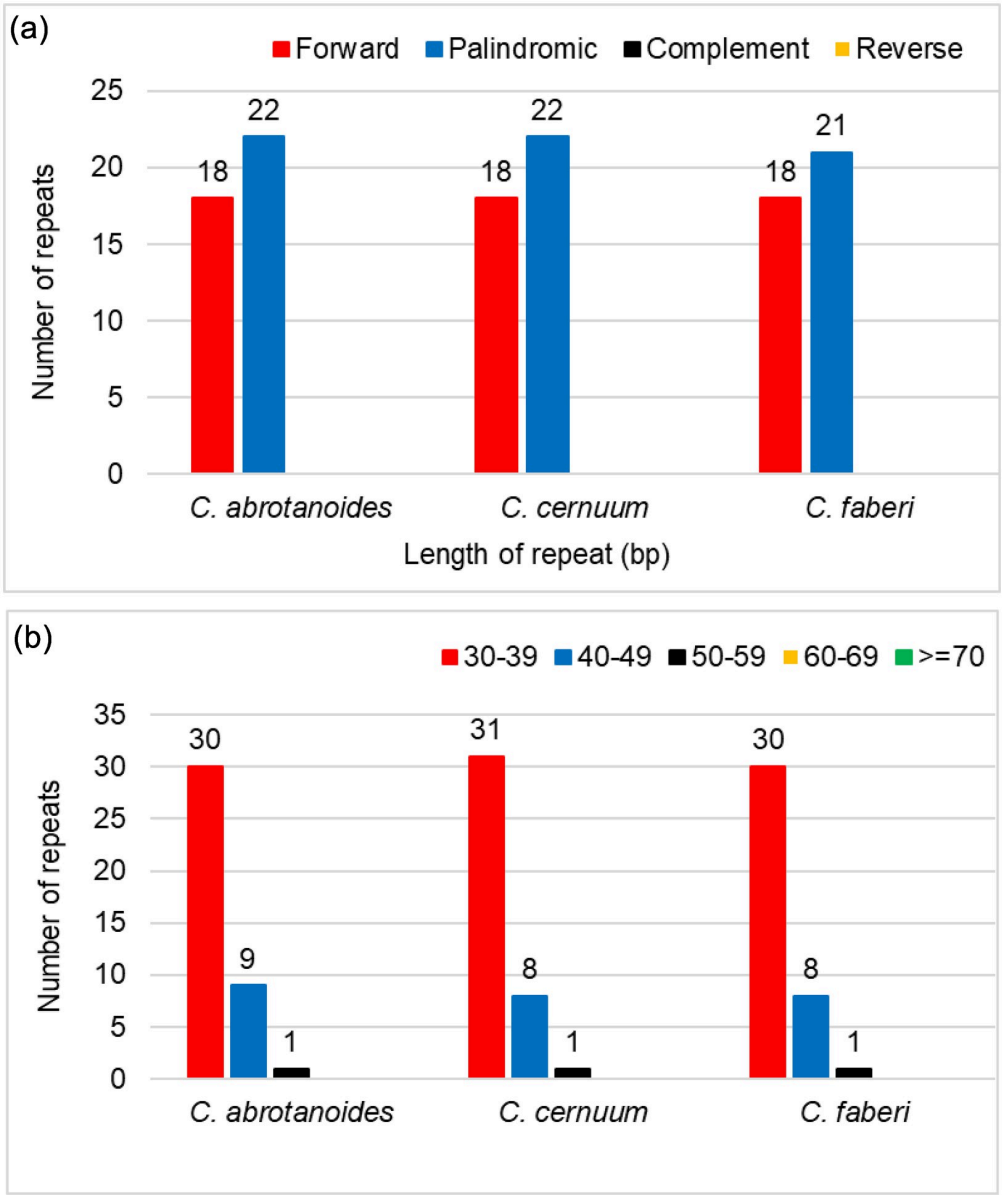
Repeat sequences in the cp genomes of three *Carpesium* species. (a) Repeat sequence types and number of repeats. (b) Number of repeat sequences of different lengths.

The repeated sequences of each cp genome were analyzed using REPuter. A total of 39–40 interspersed repeated sequences, not more than 30 bp were detected in three species of *Carpesium*, including the forward and palindromic repeats (S5 Table). The different types of repetitive sequences in the same species showed differences but not in the same type of repetitive sequences, among different species. Palindrome repeats were the most common (55%), followed by the forward repeats (45%) in *C*. *abrotanoides* and *C*. *cernuum* cp genome. Also, palindrome repeats accounted for 54%, and the forward repeats accounted for 46% in the genome of *C*. *faberi* (Fig 4a). The repeat sequence length of the majority was 30–40 bp (Fig 4b).

### IR expansion and contraction

**Fig 5:**
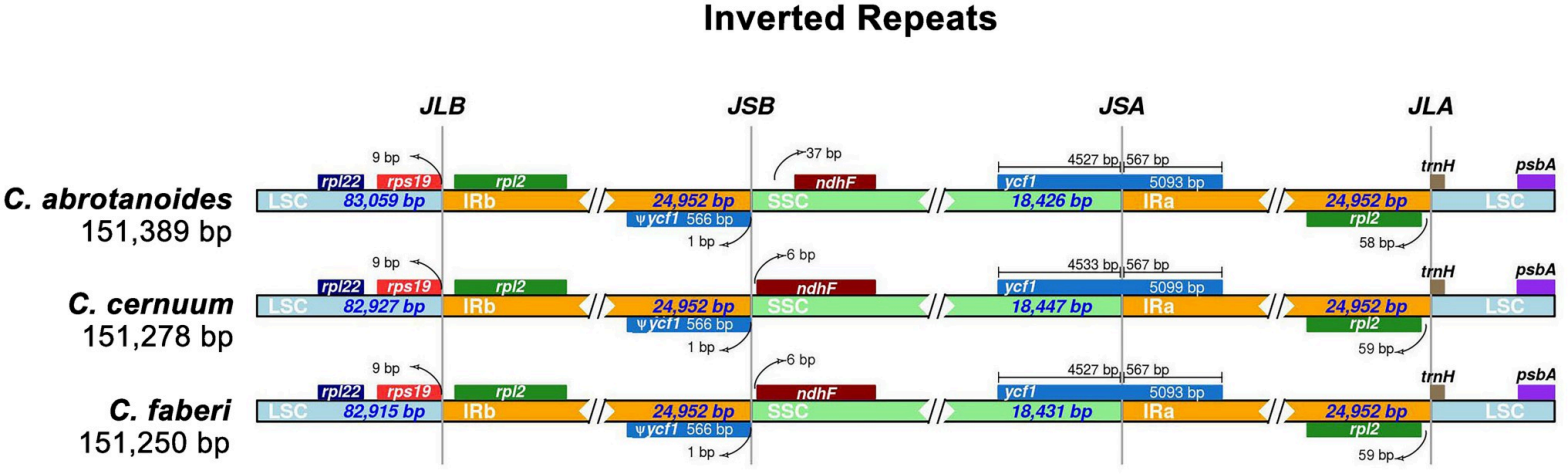
Comparative analysis of the LSC, IRs, and SSC boundary regions of the cp genomes of three *Carpesium* species.

Based on the comparison of the IR/SC boundary regions, the cp genomes of the three *Carpesium* genera showed that their expansion and contraction were similar (Fig 5). The *rpl22*, *rps19*, *rpl2*, *trnH*, and *psbA* genes were almost distributed in the LSC/IR border, whereas *ycf1* and *ndhF* genes were in the SSC/IR border. The gene *ycf1* crossed the SSC/IRa region, and the pseudogene fragment ψ*ycf1* was located at the IRb region, close to the SSC/IRb border. *ndhF* was 37 bp, 6 bp, 6 bp away from the SSC/IRb border in *C*. *abrotanoides*, *C*. *cernuum*, and *C*. *faberi*, respectively. These results suggest that *C*. *cernuum* and *C*. *faberi* are more similar than *C*. *abrotanoides*.

### Comparative genome analysis and divergence hotspot regions

**Fig 6:**
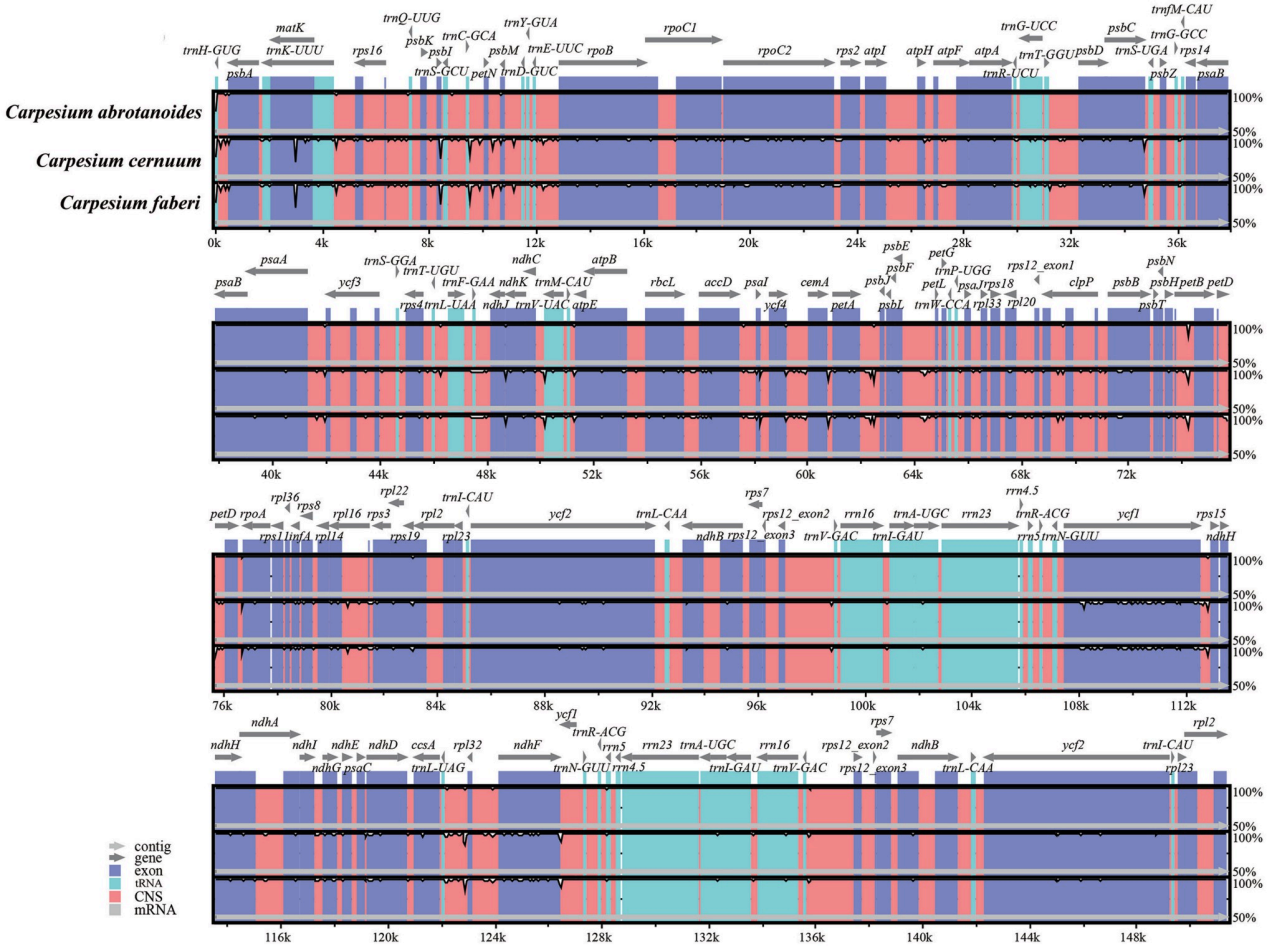
Global alignment of three cp genomes of *Carpesium* generated using Mvista with *C*. *abrotanoides* as a reference. The y-axis represents the range of identity (50%–100%). The x-axis indicates the coordinate in the cp genome. The gray arrows above the comparison represent the gene orientation and location, and different colors indicate the various regions of the genome.

**Fig 7:**
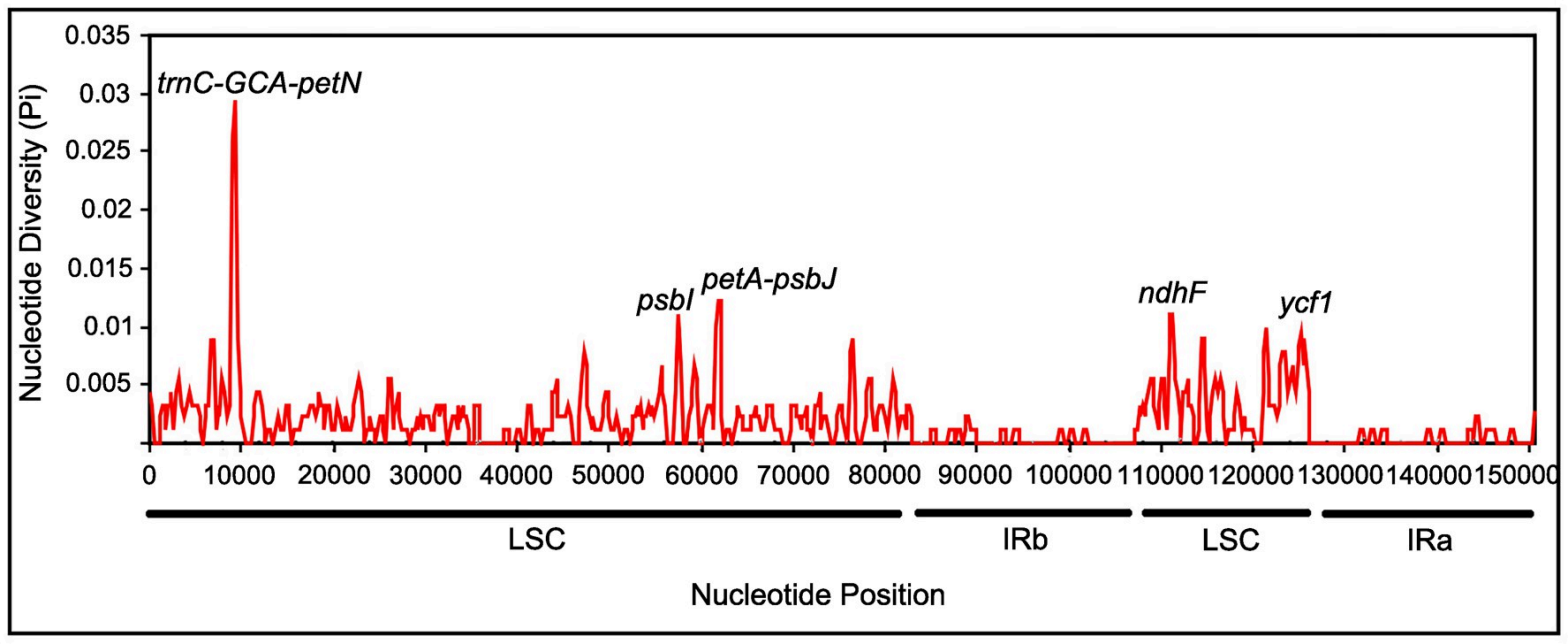
Sliding window analysis of the cp genomes of three *Carpesium* species.

The complete cp genomes of the three *Carpesium* species were compared and plotted using mVISTA by aligning the cp genomes with *C*. *abrotanoides* as the reference in elucidating the levels of sequence divergence (Fig 6). The results showed higher sequence variation in conserved non-coding sequences regions than in conserved protein-coding regions. Also, the conservation of the IR region was higher than the LSC and SSC regions, whereas the rRNA genes were highly conserved almost without variation. Furthermore, the coding regions with a large variation in the three cp genomes were *matK*, *accD*, *rpoA*, *ccsA*, *psbI*, *ndhF*, and *ycf1*, whereas the other genes had a higher degree of conservation. Variant loci in intergenic regions were significantly higher than those in the gene regions. The intergenic regions included *trnH-psbA*, *rps16-trnQ*, *trnC-petN*, *petA-psbJ*, *psbA-ycf3* etc. To clarify the variation in the higher regions, we calculated the nucleotide diversity values (pi) using DNAsp v.6.10 software (Fig 7). Five divergent loci (*trnC-GCA-petN*, *psbI*, *petA-psbJ*, *ndhF*, and *ycf1*) had a P-value ≥ 0.01, with the *trnC-GCA-petN*, *psbI*, *petA-psbJ* located in the LSC region, whereas the other loci were in the SSC region, and none being detected in the IR region. These results confirm that the LSC and SSC regions were more variable than the two IR regions.

### Phylogenetic analysis

**Fig 8:**
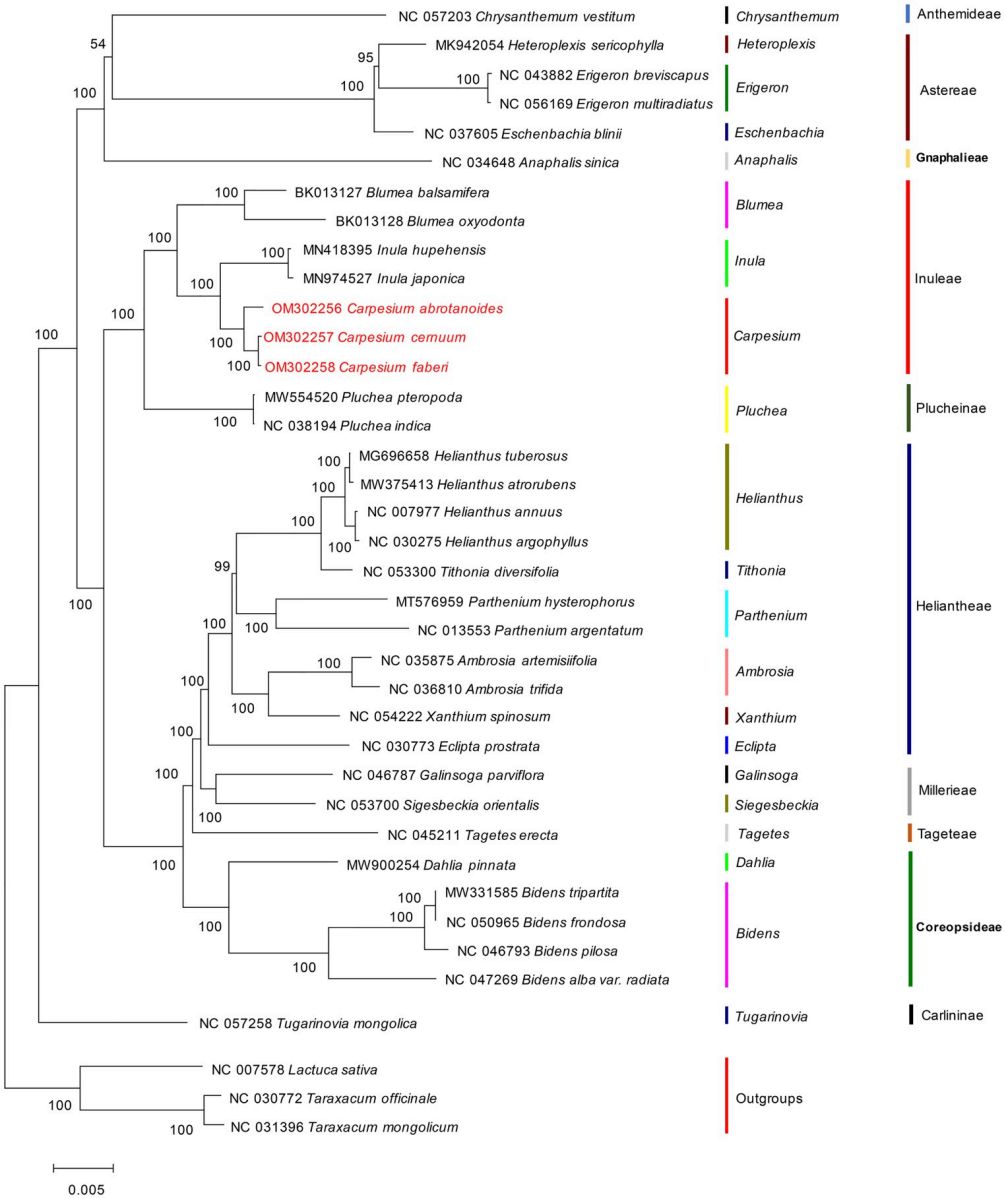
Maximum likelihood (ML) method phylogenetic tree constructed based on cp genome sequences of 38 species. The data next to each column represent bootstrap test scores. *Taraxacum mongolicum*, *Taraxacum officinale*, and *Lactuca* sativa were set as outgroups.

The phylogenetic tree was reconstructed for 38 species of Asteraceae using the best fit model TVM+F+R3. Most branch points had high bootstrap values, shown in Fig 8. The figure showed that all Asteraceae species were divided into ten subgroups (Anthemideae, Astereae, Gnaphalieae, Inuleae, Heliantheae, Millerieae, Tageteae, Coreopsideae, and Carlininae) with slight differences in the bootstrap support values of each tree topology. The genetic relationship between Inuleae and Plucheinae was closed, whereas within the Inuleae family, the genera *Blumea*, *Inula*, and *Carpesium* formed a cluster. The three species of *Carpesium* formed a monophyletic clade, which consisted of *C*. *abrotanoides* cluster, *C*. *cernuum*, and *C*. *faberi* cluster, with a bootstrap value of 100%. Additionally, the genus *Carpesium* was closely related to the Inula genus clade.

## Discussion

### Cp genome analysis

The complete cp genomes of three species of *Carpesium* were obtained using the Illumina NovaSeq sequencing technology, with comparative analysis showing highly conserved genes and structures. Similar to other sequenced angiosperm cp genomes, the *Carpesium* had a quadripartite structure typically composed of one LSC, one SSC, and two IR regions, highlighting the cp genomes with highly conserved characteristics [10]. The sizes of the cp genomes of *C*. *abrotanoides*, *C*. *cernuum* and *C*. *faberi* ranged from 151,250 bp to 151,389 bp, suggesting that the cp genome length in *Carpesium* was highly conserved, within the size range of most angiosperm cp genomes [15, 43]. The GC content distribution in the cp genomes of the three species was the same as that for other angiosperms [44, 45]. The IR regions had the highest GC content among the four other regions, followed by the LSC and SSC regions. The high GC content in the IR regions may be attributed to the presence of rRNAs (rrna4.5, rrna5, rrna23, and rrna16) with low A/T content [46].

The use of codons determines whether genetic information can be expressed correctly. It also helps to understand the molecular evolution and environmental adaptations of species and learn about the evolutionary relationships between species and genome structure, especially crucial in studying gene expression [47, 48]. The same codons were used in the three species of *Carpesium*, including 61 amino acid codons (start codon AUG) and 3 stop codons (UAA, UAG, and UGA). However, differences existed in the number and type of codons encoding the 20 amino acids that were preferentially used. Most amino acids with a preference for codons encoding, the third nucleotide contained A/U. These findings were correlated with that in other angiosperms [49, 50]. Our results also showed high similarity indices of codon usage, revealing that three species suffered from a similar environmental pressure [22].

Studies on cp genomes have shown that repetitive sequences are important for duplication, deletion, and rearrangement events [51]. Additionally, repetitive sequences are important in studying phylogeny and genome recombination [52]. The repeated analysis was performed on three cp genomes of the genus *Carpesium*, and a total of 39–40 repeat sequences were detected, mostly 30–39 bp in length. SSR or microsatellite is a commonly used class of microsatellite molecular markers [53]. The chloroplast SSRs (cp SSRs) are uniparental, simple, and possess a relatively conserved structure [54]. It also has high polymorphism, multiple alleles, and co-dominance of nuclear genomic SSR markers [55]. Therefore, it is widely used in studying population structure, genetic variation, and species identification phylogeny [56]. In this study, 37–41 SSR were detected, with single nucleotide repeat sequences (A/T), the most abundant type consistent with other angiosperms [57]. The cpSSRs also had great diversity, which may help study interspecies variety and development of molecular markers for population genetics analysis.

IR expansion and contraction are common in cp genomes and are the leading cause of cp genome size variation [58, 59]. This study found that *ndhF* was 37 bp from the SSC/IRb boundary in *C*. *abrotanoides*. In comparison, *ndhF* was only 6 bp from the SSC/IRb boundary in the other two species, which might be a reason for the longer cp genome length in *C*. *abrotanoides* than *C*. *cernuum*, and *C*. *faberi*.

The mVISTA is a common tool for comparative genomic analysis used to rapidly identify conserved regions of DNA sequences [60]. In this study, mVISTA software was used to compare and analyze the cp genomes of these three species. We found that the sequence differentiation of the cp genomes was lower, the IR region was more conserved than the SC region, and the coding region was more conserved than the non-coding region, consistent with the cp genomes of most high angiosperms [59]. The subsequent calculation of Pi values further clarified the changes in the coding region. Also, a high variation in these genes (*matK*, *accD*, *rpoA*, *ccsA*, *psbI*, *ndhF* and *ycf1*) and intergenic regions (*trnH-psbA*, *rps16-trnQ*, *trnC-petN*, *petA-psbJ*, and *psbA-ycf3*, etc) were recorded. It has also been shown that *matK*, *ycf1*, *trnH-psbA*, *rps16-trnQ*, *atpH-atpI*, and *psaA-ycf3* can be used as DNA barcodes for other plant taxa [61–64]. These highly variable regions can provide abundant and significant information for resolving the interspecific relationships of *Carpesium* in the phylogeny of the Asteraceae.

### Phylogenetic analysis

*Carpesium* is a genus attributed to the Asteraceae family, with its species similar in morphology and widely distributed. Recently, researchers have successively applied DNA sequence of cp regions (*ndhF*, *trnL-F*, *trnH-psbA*, *rps16-trnQ*, *rpl32-trnL*, *ndhF-rpl32*) and nuclear ribosomal region (ITS, ETS) for taxonomic studies, suggesting that *Carpesium* has a polyphyletic nature [65–70]. These studies are the foundation for the classification and identification of the *Carpesium*. However, the relatively short length of cp or nuclear gene sequence fragments limits phylogenetics, resulting in phylogenetic trees with low support values. Therefore, further phylogenetic classification of the genus *Carpesium* is required.

The complete cp genome is a powerful means for explaining phylogenetic relationships among species due to its rich phylogenetic information. It has been successfully used in phylogenetic studies of angiosperms [71, 72]. In this study, complete cp genomes of 38 species were used for phylogenetic analysis. The ML analysis results showed that the tested *Carpesium* formed a monophyletic lineage in phylogenetic evolution with 100% support values, closely related to genera such as *Inula*, *Blumea*, and *Pluchea*. The classical taxonomic approach places *C*. *abrotanoides* and *C*. *faberi* in the Sect. Abrotanoides and *C*. *cernuum* in the Sect. Carpesium. However, this study found that *C*. *cernuum* was more closely related to *C*. *faberi*, deviating from the traditional morphological classification method. Further study is needed to ascertain whether the traditional taxonomy is reasonable or truly shows the relationship between the species of this genus. It is impossible to resolve questions about the relationship of species under the genus *Carpesium* and the subclassification of the genus due to the availability of a few cp genome sequences of *Carpesium* and Asteraceae. More studies are therefore needed on the complete cp genome of this genus so that we can accurately analyze the affinities between the species.

## Conclusion

In this study, the cp genomes of three species of *Carpesium* (*C*. *abrotanoides*, *C*. *cernuum*, and *C*. *faberi*) were sequenced and annotated using high-throughput sequencing technology. Through bioinformatics analysis, we compared the cp genomes of these three species revealing that the structure and gene content of the cp genomes among the three *Carpesium* species were highly similar and conserved, indicating a close relationship with each other. Approximately, 40 SSR loci were identified with potentials to be used as molecular markers in studying the diversity in the genus *Carpesium*. It was also discovered that five mutation hot spots could be used to develop DNA markers suitable for the interspecies discrimination between *Carpesium*. Maximum likelihood (ML) tree analysis showed that the three *Carpesium* plants were entirely clustered into one branch and were closely related to the *Inula* plants. This study on the cp genomes of the three *Carpesium* genera provides valuable information for the species, enriches *Carpesium* cp genome data, and provides genetic resources for further species identification and phylogenetic studies of this genus.

## Supporting information

S1 Table List of species GenBank accessions numbers were used in phylogenetic analysis. (XLSX)

S2 Table The intron containing genes of the cp genomes of three *Carpesium* species and their exon and intron lengths. (XLSX)

S3 Table Codon usage in chloroplast genomes of three *Carpesium* species. (XLSX)

S4 Table SSR type and number identified in three *Carpesium* species. (XLSX)

S5 Table Repeat sequences (≥ 30bp) identified in three *Carpesium* species. (XLSX)
